# Usefulness of Double-Balloon Endoscopy in the Postoperative Gastrointestinal Tract

**DOI:** 10.1155/2011/429462

**Published:** 2011-12-11

**Authors:** Masaki Endo, Yukito Abiko, Syuhei Oana, Norihiko Kudara, Takashi Kosaka, Toshimi Chiba, Yasuhiro Takikawa, Kazuyuki Suzuki, Tamotsu Sugai

**Affiliations:** ^1^Department of Gastroenterology and Hepatology, School of Medicine, Iwate Medical University, 19-1 Uchimaru, Morioka 020-8505, Japan; ^2^Department of Diagnostic Pathology, School of Medicine, Iwate Medical University, 19-1 Uchimaru, Morioka 020-8505, Japan

## Abstract

*Background*. The small intestine has been considered to be a highly difficult organ to visualize in imaging examinations due to its anatomical location compared with the stomach and the colon. In recent years, many imaging modalities have become available, such as CT enterography, MR enterography, capsule endoscopy (CE), and double-balloon endoscopy (DBE). *Patients and Methods*. DBE was performed in the postoperative intestines of 91 patients (128 DBE examinations) at Iwate Medical University between 2004 and 2010. There were 61 male and 30 female patients, and their mean age was 69.7 years (range: 30–80 years). *Results*. A total of 124 DBE examinations were performed with endoscope insertion into the reconstructed intestines. The endoscope reached the blind end in 115 of 124 examinations, (92.7%). There were 17 patients with obscure gastrointestinal bleeding in whom 30 DBE examinations were performed. The bleeding site was identified in 12 patients (70.6%). Nine patients underwent endoscopic treatment. Hemostasis was achieved in all patients. *Conclusion*. DBE is very useful modality for the assessment and application of endotherapy to areas of the small bowel which have been altered by surgery.

## 1. Introduction

The small intestine has been considered to be a highly difficult organ to visualize in imaging examinations due to its anatomical location in comparison to the stomach and the colon. For the small intestine, the most common imaging method has been fluoroscopy. However, this method is relatively invasive, and minute lesions are difficult to visualize due to overlapping of long small intestine. Less invasive and more detailed imaging examinations are currently available, such as CT enterography [1] and MR enterography [2]. In 2000 and 2001, the first publication was made regarding two revolutionary types of endoscopy: capsule endoscopy (CE) [3] and double-balloon endoscopy (DBE) [4]. CE was developed as enteroscopy, but its applications have expanded since then to the esophagus and colon. CE is passive endoscopy unlike conventional gastrointestinal endoscopy. The patients only need to swallow a capsule endoscope body, and the examination will proceed as the endoscope moves with peristalsis. The patients experience no pain, and it has been assessed to be highly safe. The image quality is satisfactory for screening purposes. The diagnostic accuracy is the same as or better than that of CT enterography and MR enterography [5]. DBE is a groundbreaking type of endoscopy devised by Yamamoto et al. which makes the endoscopic examination and treatment of the entire small intestine possible. Each modality is useful for examining the entire small intestine. DBE is superior to other modalities because it makes it possible to perform various treatments under endoscopy.

Endoscope insertion into the postoperative intestine is considered to be the most difficult technique in endoscopic examination of the small intestine. In particular, endoscope insertion is considered to be very difficult in the afferent loop with Roux-en-Y reconstruction because of the angle and adhesion in the anastomotic site. As a result, conventional endoscopes have low rates of reaching the blind end, and such conventional methods cannot therefore be said to be standard examination methods. Observation became possible at a high rate through the use of DBE, and hemostasis, treatment of the biliary system, dilation of the narrowed area, and so forth were continuously possible, with reports related to ERCP, in particular, occasionally observed [6, 7]. However, there are not many reports regarding cases of gastrointestinal bleeding in cases of postoperative digestive tract diseases [8]. We herein report on the insertion of DBE into the postoperative digestive tract, focusing on the efficacy in cases with gastrointestinal bleeding.

## 2. Patients and Methods

### 2.1. Patients

DBE was performed in the postoperative intestines of 91 patients (128 DBE examinations) at Iwate Medical University between 2004 and 2010. In 124 examinations, oral insertion of an endoscope was performed (i.e., endoscope insertion into reconstructed intestines). There were 61 male and 30 female patients, and their mean age was 69.7 years (mean: 30–89 years). There were 9 DBE examinations involving 8 patients with Billroth II reconstruction, 91 examinations involving 62 patients with Roux-en-Y reconstruction, and 24 examinations involving 21 patients with Traverso reconstruction. There were 26 DBE examinations (17 patients) performed in order to carry out detailed examination of obscure gastrointestinal bleeding, 6 examinations (3 patients) performed for suspicion of disease of the small intestine, and 92 examinations (71 patients) for detailed examination of the biliary tract.

### 2.2. DBE Systems

The following enteroscopes can be used with the commercially available DBE system (Fuji Film Co., Japan) for small intestine: EN-450P5 (working length: 2,000 mm, distal end diameter: 8.5 mm, and working channel diameter: 2.2 mm) and EN-450T5 (working length: 2,000 mm, distal end diameter: 9.4 mm, and working channel diameter: 2.8 mm). The total length of the overtube is 1,450 mm for both enteroscopes. The shorter enteroscope EC-450BI5 is also commercially available (working length 1,520 mm, distal end diameter: 9.4 mm, and working channel diameter: 2.8 mm). In addition, we used the trial product EC-450BM5 (working length: 1330 mm, distal end diameter: 9.4 mm, and forceps working diameter: 2.8 mm). EN-450P5 was used when DBE was performed only for examinations, but EN-450T5 was used when treatment, such as hemostasis, was necessary. In some cases, endoscopic treatments were planned, such as hemostasis near the blind end and biliary duct treatment. If the afferent loop was the target site to be reached, such as when performing the aforementioned treatments, then the shorter enteroscope was used as much as possible to match the working length of the instruments.

### 2.3. DBE Technique

In patients with Billroth II reconstruction or Traverso reconstruction, an endoscope can often be inserted without any difficulty if its direction is correct. There are a few points of caution for patients who have undergone Roux-en-Y reconstruction. First, the size of the remnant stomach can become a problem. In total gastrectomy cases without a remnant stomach, the course is relatively straight up to the site of the jejunoduodenostomy. Therefore, endoscope insertion can be accomplished without any problems. For patients with a remnant stomach, a balloon and overtube of DBE can be used to immobilize the remnant stomach and the intestine in a straight position, and the endoscope can then be advanced to the anastomotic site. Therefore, DBE is very useful in such patients. Care should be taken concerning the insufflation of air during endoscope insertion, and it is desirable to insufflate to the minimum necessary extent. For this reason, the endoscope is sometimes advanced distally without being able to verify the anastomotic site. In clinical practice, the identification of the anastomotic site is accomplished by trying to identify any branching of the intestine, while carefully noting any dilation of the lumen and the multidirectionality of circular folds. The important point is to obtain as much information as possible from the operative records, unless the procedure is emergency endoscopy. The anastomotic site can be located faster if the operative records are reviewed in advance to determine the distance between either the gastrojejunostomy or esophagojejunostomy site and the jejunojejunostomy site.

After the jejunojejunostomy site is identified, one must determine which direction of the branch is the Roux-en-Y loop to advance the endoscope further toward the duodenum. First, the presence of bile should be verified to determine the desired direction. However, bile is also present in the intestine distal to the branching site of the Roux-en-Y loop. In patients with obstructive jaundice, bile itself is absent in the intestine. Therefore, it is difficult to identify the Roux-en-Y loop by the presence of bile alone. When the branching site is reached, it is more practical to insert the endoscope several dozen centimeters into one branch and then use fluoroscopy to verify the direction in which it is advancing.

When conventional endoscopes are used, a sharp branching angle of the Roux-en-Y loop can cause difficulty of endoscope insertion. The angle can be made less acute to some extent by performing balloon dilation near the branching site. In many cases, the angle can be made less acute by pulling the endoscope while grasping the intestine with a balloon. At this time, endoscope insertion can be difficult even for DBE if there is no change in intestinal course in fluoroscopy or if some abnormal hardness is felt through the endoscope. If so, the endoscope should not be forced. The same caution is also required when an assistant inserts the overtube.

For an endoscopic examination, we premedicate our patients with 0.1-0.2 mg/kg of midazolam and 15 mg of pentazocine. If they are still alert or complain of any pain, then the doses are increased accordingly. Their vital signs are monitored carefully during the examination using a pulse oximeter, sphygmomanometer, and ECG monitor.

## 3. Results

### 3.1. Reaching the Blind End

An enteroscope was inserted into the postoperative intestines, and it reached the blind end in 115 of 124 DBE examinations (92.7%). The blind end was reachable in 8 of 9 examinations (88.9%) among the patients with Billroth II reconstruction, in 83 of 91 examinations (91.2%) among the patients with Roux-en-Y reconstruction, and 24 of 24 examinations among the patients with Traverso reconstruction (Table 1).

This study used standard enteroscopes P5 and T5 (working length of 2,000 mm) and the short types of enteroscopes BI5 and BM5. When the standard types and short types were compared, the blind end was reachable in 32 of 36 examinations (88.8%) using the standard types and in 84 of 89 examinations (94.4%) using the short types. This result indicated no substantial difference between the two types.

The time required to reach the blind end was 32.7 min (range: 5–60 min) for patients with Billroth II reconstruction, 34.3 min (range: 5–140 min) in patients with Roux-en-Y reconstruction, and 25.2 min (range: 2–64 min) in patients with Traverso reconstruction.

### 3.2. Complications

Accidents associated with endoscope insertion occurred in 4 examinations (3.2%) and consisted of gastrointestinal perforation and retroperitoneal emphysema. There was no obvious perforation during either the 2 examinations in patients with Billroth II reconstruction or 2 examinations in patients with Roux-en-Y reconstruction. However, they showed retroperitoneal emphysema on postoperative X-rays. All of these patients were successfully managed by conservative treatment.

### 3.3. ERCP Using a DBE

DBE was carried out on 92 subjects, 71 cases with the purpose of examining the biliary tract. The rate of reaching a blind end was 93.5%, and 5 cases with Roux-en-Y reconstruction were unreachable. Cholangiography was successful in 77 of 83 cases excluding 9 cases that were unreachable and those for the purpose of examining the pancreas. Endoscopic therapy was carried out on 59 cases, endoscopic sphincterotomy (EST) was carried out on 16 cases, endoscopic papillary balloon dilatation (EPBD) was carried out on 16 cases, and dilation for anastomotic stenosis after choledocojejunostomy was carried out on 5 cases; regarding others, endoscopic nasobiliary drainage (ENBD) and endoscopic biliary drainage (EBD) were carried out.

### 3.4. Obscure Gastrointestinal Bleeding

In 17 patients with obscure gastrointestinal bleeding, 30 DBE examinations were performed. Oral and anal endoscope insertion was performed in 4 patients, and oral insertion alone was performed in 13 patients. The bleeding site was identified in 12 patients (70.6%). The bleeding site was located between the anastomotic site and blind end in 10 patients (58.8%). Endoscopic treatment was performed in a total of 9 patients using argon plasma coagulation in 4 patients, hemostatic clip in 4 patients, and hemostatic forceps in 1 patient. Hemostasis was achieved in all patients. However, one patient had rebleeding at the same site, while two patients had rebleeding from another site, and these patients all underwent additional endoscopic treatment. One of these patients had multiple rebleeding from different sites, so this patient subsequently underwent surgical treatment. No treatment-associated accidents were observed (Table 2).

## 4. Case Presentation

### 4.1. Case 7

**Figure 1:**
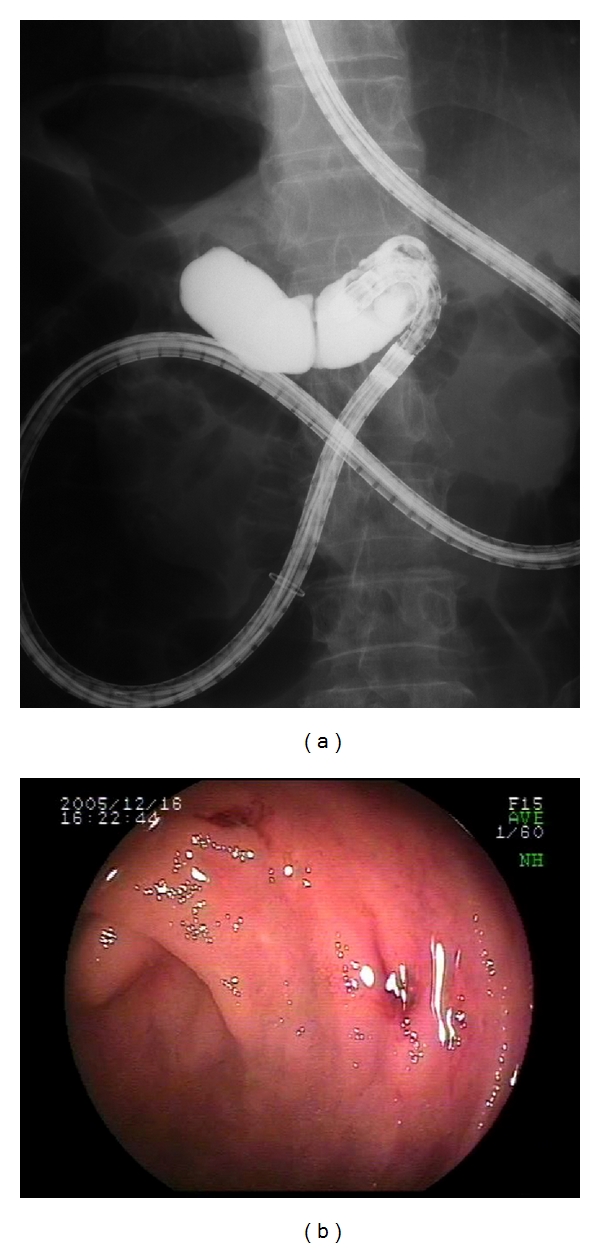
(a) Confirmation that the blind end was reached using fluoroscopy; (b) mucosal atrophy and angioectasias were found in the blind end.

The patient was a 73-year-old male. At 66 years of age, he underwent a resection of the pancreatic body with Roux-en-Y reconstruction for intraductal papillary mucinous neoplasm. He was referred to our hospital for further examination of obscure gastrointestinal bleeding. DBE (with EN-450T5) was performed at our hospital. The time required to reach the blind end was 30 minutes. Multiple angioectasias (Yano classification [9] Ia, Ib) and mucosal atrophy were observed in the region of the blind end. Hemostasis was achieved by endoscopic clipping (Figures 1(a) and 1(b)).

### 4.2. Case 8

**Figure 2:**
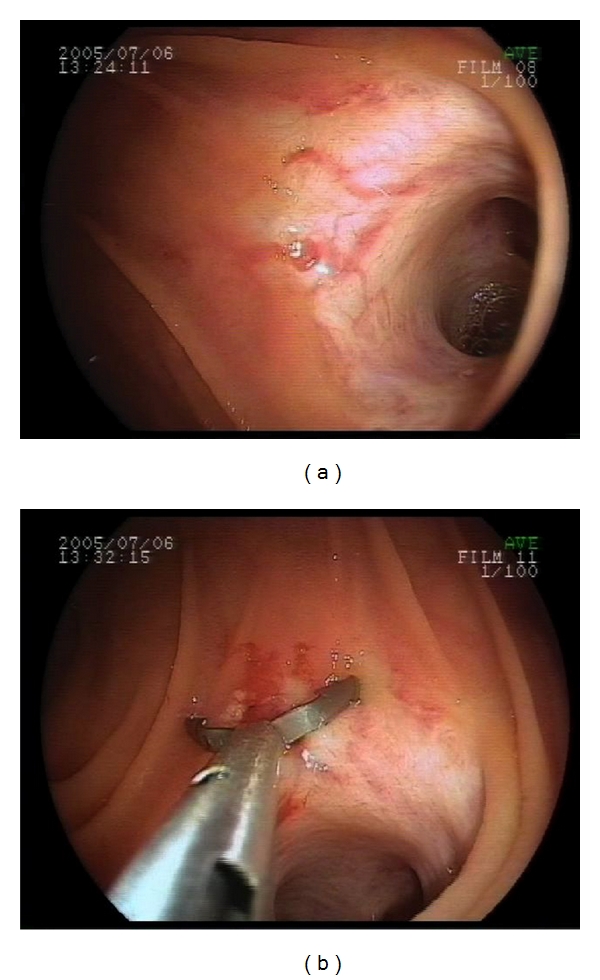
(a) Angioectasia was found at the choledochojejunostomy site. Bleeding was found after washing with water, and this site was determined to be the bleeding site. (b) Endoscopic clipping was performed.

The patient was a 63-year-old male. At 50 years of age, he underwent pylorus-preserving pancreaticoduodenectomy with Traverso reconstruction for duodenal ampullary carcinoma. He was referred to our hospital to undergo further examination of obscure gastrointestinal bleeding. DBE (with EN-450T5) was performed at our hospital. Angioectasia (Yano classification Ib) was found at the choledochojejunostomy site. Hemostasis was achieved by endoscopic clipping, and there was no subsequent bleeding (Figures 2(a) and 2(b)).

### 4.3. Case 9

**Figure 3:**
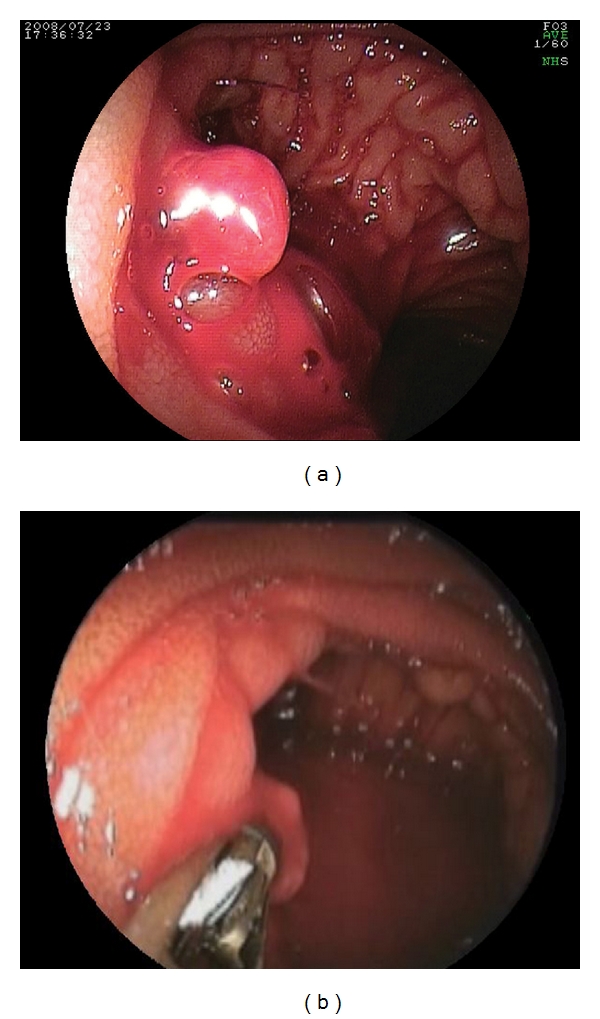
(a) A pulsatile, exposed vessel was found at the anastomotic site of the afferent loop; (b) Hemostasis was achieved using hemostatic forceps.

The patient was a 37-year-old female. She underwent living-donor liver transplantation for primary sclerosing cholangitis. She had passed tarry stool for 8 days postoperatively. Upper and lower gastrointestinal examinations were performed, but the bleeding site was not identified, so DBE (with EN-450T5) was performed. A pulsatile, exposed vessel was found at the anastomotic site of the afferent loop, and hemostasis was achieved using hemostatic forceps. No rebleeding was observed (Figures 3(a) and 3(b)).

## 5. Discussion

The small intestine has been considered to be a difficult organ to visualize by imaging examinations due to its anatomical location. CT enterography and MR enterography have revolutionized imaging with a good diagnostic performance and noninvasive examinations. These imaging methods can also be used to examine postoperative intestines. Prior to the development of these new modalities, reoperation was unavoidable in many cases with complications such as bleeding and stricture of the anastomotic site or blind end, and tumor recurrence. In particular, bleeding sites are difficult to identify, so the same examination method was used repeatedly, thus resulting in a delay of treatment [10].

We previously reported on biliary endoscopic treatments using a pediatric colonoscope to reach the blind end in cases with Roux-en-Y reconstruction [11]. However, we were not satisfied with the success rates at which the colonoscope reached the blind end and therefore attempted to develop a new device. The working lengths of double-balloon endoscopes are not made excessively long relative to the length of the small intestine. The enteroscope advances to a deeper area by inflating balloons attached to the enteroscope and overtube to grasp the inside of the intestine and then deflating them in order to advance. In the present study, the blind end was reachable in 91.2% of the cases. Shimatani et al. reported that it was reachable in 95.0% of their cases [12]. One factor that could hinder the advancement of the endoscope is malignancy as the reason for any previous surgery. Namely, adhesion caused by extensive lymph node dissection can affect the advancement of the scope. An endoscope can be successfully advanced in a high percentage of patients who underwent surgery, such as bariatric surgery, for benign disease [13, 14]. If an endoscope can reach the lesion, then hemostasis and dilatation can be performed just as in other areas of the gastrointestinal tract.

In a paper related to obscure gastrointestinal bleeding, Gerson et al. [15] reported that vascular lesions were the most common cause of hemorrhaging. However, in Japan, Shinozaki et al. [16] and Fujita et al. [17] both reported that ulcerative lesions were common. In this paper, the result was 6 cases of vascular lesion, 5 cases of ulcerative lesions, and 1 case of neoplastic lesion. It was distinctive in that, of these, it existed between the anastomotic part and the blind end in 10 cases. It was believed that afferent loop was due to the presence of bile and/or pancreatic juice and the fact that bacterial flora differs from normal inner intestines. Regarding the anastomotic part, neovessels were also involved in hemorrhaging at the early and later stages following surgery.

Kim et al. [18] also carried out DBE on 16 cases of postoperative digestive tract diseases and reported that lesions were present in the afferent loop in 2/3 of cases confirmed upon diagnosis, which was believed to be a similar result as our research.

Regarding procedural accidents related to DBE, Mensink et al. [19] reported they were found in 0.8% of diagnostic DBE and 4.3% of therapeutic DBE upon a multicenter survey among 2,362 cases. In this research, procedural accidents related to insertion were observed in 4 cases. In a paper related to the postoperative digestive tract, Parlak et al. [20] found perforations in 1 of 14 cases (7.1%). Shimatani et al. found procedural accidents in 5 of 103 cases (4.9%) and reported that of these, 3 cases involved perforation or pneumoretroperitoneum. Though no obvious perforations were confirmed in any of our cases, pneumoretroperitoneum was observed. Though requiring fasting for several days, it lacked symptoms such as pain and fever, and though emphysema was observed upon CT, effusion did not emerge, and conservative medical treatment was possible in all cases. However, for cases in which obvious perforations are confirmed, the opportunity for surgical treatment should also not be missed.

Reconstruction for cases of procedural accidents included 2 cases of Billroth II reconstruction and 2 cases of Roux-en-Y reconstruction. Regarding Billroth II reconstruction, many cases are insertable by conventional endoscopy, so only cases in which insertion is difficult, such as high-adhesion cases, undergo reexamination by DBE. Thus, it is believed that the outcome of this report shows no differences in insertion time, rate to reach a blind end, and rate of procedural accidents as that of Roux-en-Y reconstruction. A more careful manipulation was necessary, understanding that it is a case in which insertion is difficulty. The 2 cases of Roux-en-Y reconstruction were suspected of carcinomatous peritonitis and adhesion after extensive lymph node dissection. In such cases, giving priority to other examinations should be considered as well.

## 6. Conclusion

Today, many methods for examination have become possible for diseases of the small intestines. Of these, DBE allows for investigation and treatment even for cases of postoperative digestive tract, indicating it as a useful treatment.

## Figures and Tables

**Table 1:** Reconstruction methods and the results of DBE insertion.

	Reaching the blind end		Time to reach the blind end	Complication	
	*n*	%	min	*n*	%
Billroth-II	8/9	88.9	32.7	2/9	22.2
Roux-en-Y	83/91	91.2	34.3	2/91	2.2
Traverso	24/24	100	25.2	0/24	0

Total	115/124	92.7	32.2	4/124	3.2

**Table 2:** Summary of the cases and results of DBE.

Case	Age/sex	DBE findings	Location of lesions	Endoscopic treatment	Complication
1	62 F	Erosion	Ileum	APC	No
2	61 M	Angioectasia	Colon	APC	No
3	59 F	Angioectasia	Afferent loop anastomosis	APC	No
4	63 M	Erosion	Afferent loop	APC	No
5	84 F	Ulcer	Afferent loop anastomosis	Clip	No
6	89 F	Ulcer	Afferent loop anastomosis	Clip	No
7	73 M	Angioectasia	Afferent loop	Clip	No
8	63 M	Angioectasia	Bilioenteric anastomosis	Clip	No
9	37 F	Exposed vessel	Afferent loop anastomosis	Hemostatic forceps	No
10	69 F	Tumor	Bilioenteric anastomosis	None	No
11	52 M	Ulcer	Afferent loop anastomosis	None	No
12	66 M	None		None	No
13	72 M	None		None	No
14	71 F	None		None	No
15	79 M	None		None	No
16	66 M	Angioectasia	Bilioenteric anastomosis	None	No
17	76 M	None		None	No
